# A nationwide survey of antimicrobial dispensation practices in pharmacies and bodegas in the Dominican Republic

**DOI:** 10.1017/ash.2022.314

**Published:** 2022-10-25

**Authors:** Alfredo J. Mena Lora, Rita Rojas-Fermin, Bismarck Bisono, Marcos Almonte, Susan C. Bleasdale

**Affiliations:** 1 University of Illinois at Chicago, Chicago, Illinois, United States; 2 Hospital General Plaza de la Salud, Santo Domingo, Dominican Republic; 3 Mayo Clinic, Rochester, Minnesota, United States; 4 Baptist Medical Center, Trenton, New Jersey, United States

## Abstract

In many developing countries, antimicrobials are available without prescriptions in pharmacies and stores. We performed a survey to describe antimicrobial availability, training, and use recommendations for common symptoms in the Dominican Republic. Pharmacy recommendations varied, whereas aminopenicillins are routinely recommended at bodegas. Frontline staff are gatekeepers and potential targets for stewardship education.

The wide availability and indiscriminate use of antimicrobials contributes to antimicrobial resistance (AMR) and threatens their continued effectiveness. In the United States, antimicrobial use in ambulatory settings is commonly guideline discordant or without indication.^1^ Antimicrobial use in ambulatory settings is also a problem in low- and middle-income countries (LMICs), where antimicrobials can be purchased without prescriptions and are available in pharmacies and stores.^2,3^ These factors may be contributing to the steady rise of AMR in LMICs in the past decade.^4^

In Latin America, AMR is widespread and varies by region. Extended-spectrum β-lactamase (ESBL) organisms are common, estimated at 11%–25% of *Escherichia coli* and 45%–53% of *Klebsiella pneumoniae*.^5^ Carbapenem resistance is also on the rise, with growing presence in Colombia and Brazil.^6^ In the Dominican Republic, >30% of *E. coli* have resistance patterns consistent with ESBL.^7^ Thus, understanding antimicrobial dispensation practices in LMICs like the Dominican Republic is essential for the development of mitigation strategies and public policy. We conducted a nationwide survey to describe antimicrobial availability, staff training, and dispensation practices in pharmacies and stores in the Dominican Republic.

## Methods

### Study design and setting

We conducted a cross-sectional survey of staff that dispense antimicrobials in pharmacies and bodegas. Sites were randomly selected from cities across the Dominican Republic including Santo Domingo, Santiago de los Caballeros, Puerto Plata, Higuey, La Romana, Bani, and Azua. Bodegas, also known as “colmados” or “mini-markets,” are small neighborhood stores where dry goods, food, household items, and over-the-counter (OTC) medications are sold. Bodegas often take orders from clients via telephone, Internet, or telephone applications and deliver goods via motorcycle courier to locations within their neighborhood. Bodegas may sell OTC medications, including antimicrobials. Pharmacies in the Dominican Republic sell antimicrobials with or without prescriptions and may also deliver via motorcycle courier to locations within their neighborhood. It is legal to buy and sell antimicrobials in the Dominican Republic without prescriptions.^8^

### Cross-sectional survey

Pharmacies and bodegas were identified in Google maps and randomly selected. Participants were recruited via telephone after verbal consent (Supplement 1). Each store completed a one-time survey. Multiple pharmacies and bodegas were surveyed between March 1, 2019, and November 30, 2019. The survey consisted of a standardized script translated to Spanish via a translation service (Supplement 2). Surveys were performed by a native Spanish speaker. Participants were asked what antimicrobial they would recommend and dispense for adults requesting antimicrobials for specific symptoms (Table 1). Each question represented a scenario that would trigger an antimicrobial use recommendation and dispensation from the pharmacy or bodega if asked by a client. Questions regarded availability of antimicrobials at the day of the survey, use of prescriptions, prior training on antimicrobials, and purchase and delivery options. Data were collected in a deidentified fashion and were grouped by general geographic neighborhood without store or provider identifiers. These data were tabulated in a Microsoft Excel spreadsheet (Microsoft, Redmond, WA). The University of Illinois at Chicago Institutional Review Board approved this study.

**Table 1.** Survey questions on antimicrobial use recommendations by symptom category

Symptom category	Survey questions on antimicrobial use recommendations in English
**Febrile illness**	If someone asks for an antibiotic for fever, what antibiotic would you recommend?
	If someone asks for an antibiotic for influenza-like illness or a cold, what antibiotic would you recommend?
	If someone asks for an antibiotic for a cough, what antibiotic would you recommend?
	If someone asks for an antibiotic for a pneumonia, what antibiotic would you recommend?
**Throat symptoms**	If someone asks for an antibiotic for throat pain, what antibiotic would you recommend?
**Urinary symptoms**	If someone asks for an antibiotic for pain urinating or a urinary tract infection, what antibiotic would you recommend?
**Gastrointestinal symptoms**	If someone asks for an antibiotic for diarrhea, what antibiotic would you recommend?

## Results

**Fig. 1.**
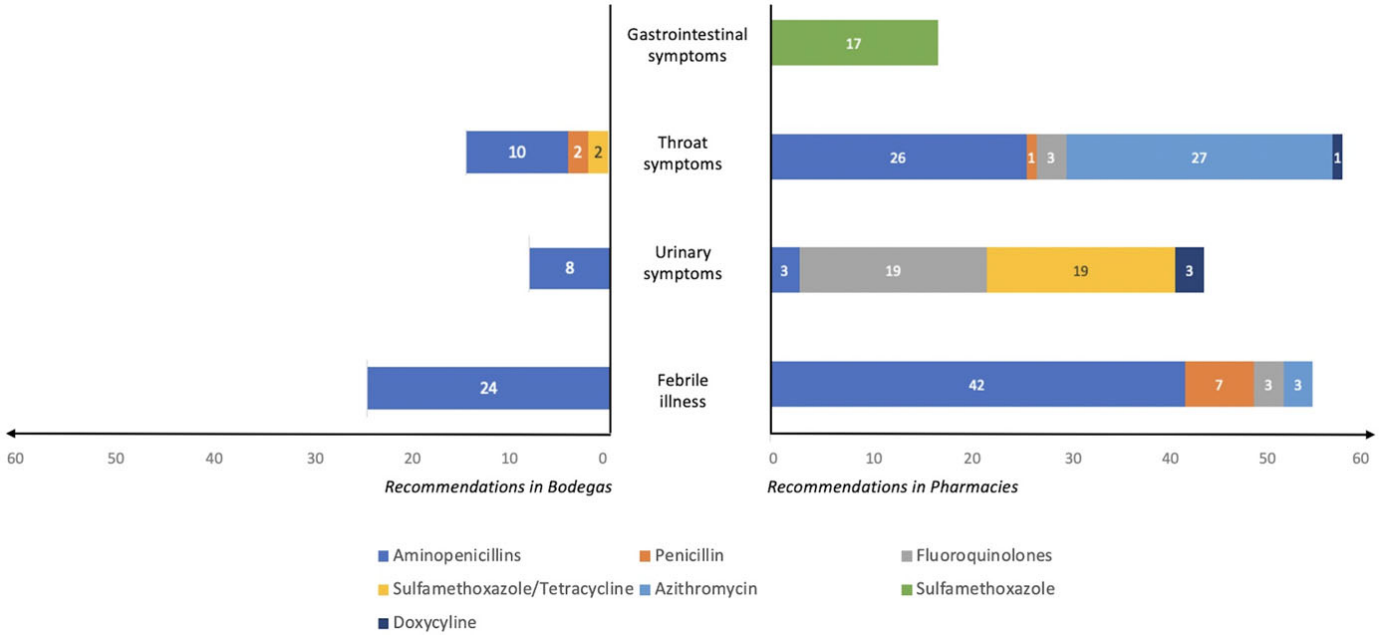
Antimicrobial use recommendations by symptom category in pharmacies and bodegas.

In total, 125 stores were invited to participate during the study period; 34 pharmacies and 48 bodegas agreed to participate. In total, 174 antimicrobial use recommendations were given in pharmacies and 46 were given in bodegas for different clinical scenarios, representing 73% and 14% of the clinical scenarios presented, respectively. No answers or recommendations were given in 64 of the clinical scenarios asked at pharmacies and 290 of those presented at bodegas. The most common antimicrobials recommended were aminopenicillins, representing 71 recommendations (41%) in pharmacies and 42 recommendations (95%) in bodegas (Fig. 1). In pharmacies, antimicrobial use recommendations varied by syndrome, with 42 recommendations (80%) for aminopenicillins (most commonly for febrile illness), 19 recommendations (43%) for fluoroquinolones and 19 recommendations (43%) for sulfamethoxazole-tetracycline (ie, the most common for urinary complaints), 27 recommendations (46%) for azithromycin (the most common for throat complaints) and 17 recommendations (100%) for sulfamethoxazole (the most common for gastrointestinal complaints) (Fig. 1). In bodegas, 42 aminopenicillin recommendations (95%) were given; it was the most common recommendation for all symptoms. Staff had received prior training on antimicrobials in 61% of pharmacies and 0% of bodegas. Antimicrobials were available for delivery via telephone order in 80% of pharmacies and 90% of bodegas. All sites sold antimicrobials with and without a prescription. No antimicrobials were available via online delivery applications.

## Discussion

Antimicrobials were readily available in pharmacies and bodegas surveyed across the Dominican Republic, both with and without prescriptions. Antibiotic use recommendations differed between pharmacies and bodegas, where no training on antimicrobials was reported. Pharmacies gave recommendations for more scenarios presented than bodegas (84% vs 12%), and the recommendations in pharmacies were more nuanced. Although bodega staff had no answers or recommendations for most clinical scenarios (88%), there were no barriers to purchasing antimicrobials, and clients likely purchased antimicrobials with less guidance in bodegas, where staff are untrained. In pharmacies, different antimicrobials were recommended by symptom; in bodegas, aminopenicillins represented almost all recommendations. Removing the OTC availability of antimicrobials and limiting dispensation to pharmacies may improve antimicrobial use. In the interim, training for individuals that dispense antimicrobials and patients who seek them should be scaled up.

Aminopenicillins were the most recommended antimicrobials. This overdistribution may be contributing to high rates of ESBL-producing organisms in the Dominican Republic.^7^ A review of 6,299 outpatient *E. coli* isolates revealed that >30% had a pattern consistent with ESBL-producing organisms, and resistance to fluoroquinolones and trimethoprim-sulfamethoxazole was present in >54% of samples. AMR was highest in lower-income communities.^7^ These communities may have less access to healthcare and may be prone to self-prescribing antimicrobials, which subsequently drives AMR.

Self-medication with antimicrobials has been reported in ethnic and low-income communities in the United States.^9,10^ These antimicrobials are usually purchased at local stores without prescriptions or are obtained via family members who travel abroad where antimicrobials are more easily purchased. A survey of 219 adults in a South Carolina Latinx community found that >16.4% brought antimicrobials from abroad and 19.2% obtained antimicrobials in the United States without a prescription.^10^ Interestingly, 30.6% believed antimicrobials should be available without prescriptions. A survey of 101 stores in 3 New York City neighborhoods revealed that antimicrobials were available in all the surveyed stores in Latinx-predominant neighborhoods and none of the stores that served other ethnic groups.^9^ In the 2020 census, Americans of Dominican descent represented the largest group among Hispanics in New York City, surpassing Puerto Ricans. Antimicrobial practices may reflect perceptions and practices from countries of origin.

Frontline staff in pharmacies and bodegas are gatekeepers for antimicrobial use and may be an important target for antimicrobial stewardship initiatives. Further studies are needed to develop a deeper understanding of antimicrobial use in LMICs. Furthermore, both in the United States and in LMICs, understanding factors that drive antibiotic pressure in the community is important for the development of education and antimicrobials stewardship strategies that may help reduce excess use.

## Supplementary material

For supplementary material accompanying this paper visit https://doi.org/10.1017/ash.2022.314. click here to view supplementary material
