# Supplementary material for: A nationwide survey of antimicrobial dispensation practices in pharmacies and bodegas in the Dominican Republic
Source: Antimicrob Steward Healthc Epidemiol. 2022 Oct 25;2(1):e173. doi: 10.1017/ash.2022.314 (PMC9726472; doi:10.1017/ash.2022.314)
Supplement: Supplementary file 1 [file S2732494X2200314Xsup001.docx]

**SUPPLEMENTARY MATERIALS**

**Supplement 1. Verbal consent in English and Spanish**

*Hello! I am a researcher from the University of Illinois at Chicago. I am interested in antibiotics and how they are sold. Would you mind if I ask you a few questions on antibiotics? Your answers will help us understand how antibiotics are used in your neighborhood. We will not identify you or your store, only the general neighborhood area. Let me know if you have any questions and if you are willing to participate. Thank you!*

**Supplement 2. Survey tool in English and Spanish**

| Survey tool (English) | Survey tool (Spanish translation) |
| --- | --- |
| What antibiotics do you have available? | Tipos de antibióticos disponibles |
| If someone asks for an antibiotic for fever, what antibiotic would you recommend? | Si alguien pide un antibiótico para fiebre, que antibiótico recomendarías? |
| If someone asks for an antibiotic for a cough, what antibiotic would you recommend? | Si alguien pide un antibiótico para tos, que antibiótico recomendarías? |
| - If someone asks for an antibiotic for influenza-like illness or a cold, what antibiotic would you recommend? | Si alguien pide un antibiótico para la gripe, que antibiótico recomendarías? |
| If someone asks for an antibiotic for pain urinating or a urinary tract infection, what antibiotic would you recommend? | Si alguien pide un antibiótico para dolor de orina o infección de orina, que antibiótico recomendarías? |
| If someone asks for an antibiotic for throat pain, what antibiotic would you recommend? | Si alguien pide un antibiótico para dolor de garganta, que antibiótico recomendarías? |
| If someone asks for an antibiotic for a pneumonia, what antibiotic would you recommend? | Si alguien pide un antibiótico para neumonia, que antibiótico recomendarías? |
| If someone asks for an antibiotic for diarrhea, what antibiotic would you recommend? | Si alguien pide un antibiótico para diarrhea, que antibiótico recomendarías? |
| Is there any training or education on antibiotics for those who sell them? | Reciben algún curso o educación sobre antibióticos los que los venden? |
